# Quality of Antenatal Care and Obstetrical Coverage in Rural Burkina Faso

**DOI:** 10.3329/jhpn.v28i1.4525

**Published:** 2010-02

**Authors:** L. Nikiema, Y. Kameli, G. Capon, B. Sondo, Y. Martin-Prével

**Affiliations:** ^1^ Institute of Research in Health Sciences, 03 BP 7192 Ouagadougou 03, Burkina Faso; ^2^ Institute of Research for Development, UR106 «Nutrition, Alimentation, Sociétés», Centre Collaborateur de l'OMS pour la Nutrition-911 avenue Agropolis–BP 64501–34394 Montpellier Cedex 5, France; ^3^ IRD–UR106-01 BP 182-Ouagadougou 01, Burkina Faso

**Keywords:** Antenatal care, Cross-sectional studies, Deliveries, Developing countries, Maternal health services, Obstetric care, Quality of care, Burkina Faso, Africa

## Abstract

Improving maternal health is one of the Millennium Development Goals of the United Nations. Despite the efforts to promote maternal and neonatal care to achieve this goal, the use of delivery care remains below expectations in Burkina Faso. This situation raises the question of the quality of care offered in maternity wards. The aim of this study was to identify primary healthcare facility and antenatal care characteristics predictive of an assisted delivery in rural Burkina Faso. A cross-sectional study was carried out in Gnagna province (North-East Burkina Faso) in November 2003. The operational capacities of health facilities were assessed, and a non-participating observation of the antenatal care (ANC) procedure was undertaken to evaluate their quality. Scores were established to summarize the information gathered. The rate of professional childbirth (obstetrical coverage) was derived from the number of childbirths registered in the health facility compared to the size of the population. The established scores were related to the obstetrical coverage using non-parametric tests (Kendall). In total, 17 health facilities were visited, and 81 antenatal consultations were observed. Insufficiencies were observed at all steps of ANC (mean total score for the quality of ANC=10.3±3.0, ranging from 6 to 16, out of a maximum of 20). Health facilities are poorly equipped, and the availability of qualified staff remained low (mean total score for the provision of care was 22.9±4.2, ranging from 14 to 33). However, these scores were not significantly related to the rate of professional childbirth (tau Kendall=0.27: p=0.14 and 0.01, p=0.93 respectively). The ability of the primary health centres to provide good antenatal care remains low in rural Burkina Faso. The key factors involved in the limited use of professional childbirth relating to maternal health services may be the quality of ANC.

## INTRODUCTION

Maternal mortality remains a major public-health issue in developing countries. According to the World Health Organization (WHO), 536,000 women die every year in the world from causes relating to pregnancy, childbirth, or postpartum. Ninety-nine percent of these deaths occur in developing countries (1). The majority of maternal deaths could be avoided if women had access to quality medical care during pregnancy, childbirth, and postpartum (1).

The existence of properly-developed medical facilities and the availability of qualified personnel for childbirth are certainly key factors if maternal mortality is to be reduced. Only 62% of childbirths are assisted by qualified people in developing countries (2). The process of medically-assisted childbirth begins with antenatal care (ANC) attendance, which is the first contact with health services during pregnancy and on which the continuation of prenatal care mainly depends. Moreover, antenatal follow-up plays an important role in reducing perinatal morbidity and mortality.

ANC helps establish contacts with pregnant women at the peripheral level so as to detect, treat, or prevent infections likely to cause after-effects or death of mothers (3, 4).

Overall, 15% of pregnant women are estimated to suffer from life-threatening complications which could have been detected during antenatal consultations (5). Some authors reported that ANC alone could reduce the maternal death rate by more than 20% (5, 6), provided that ANC is of good quality and regularly attended by pregnant women. Because of the importance of maternal health for development, the WHO and the United Nations Population Fund jointly launched the Safe Motherhood Initiative in Nairobi in 1987. As a result, significant investments were made in developing countries to improve prenatal care, with real success, particularly in sub-Saharan Africa and especially in urban areas (1, 7). However, maternal mortality has remained stagnant in some countries (1). More recently, to encourage actions aimed at reducing maternal death rates, improvement of maternal health was included in Objective 5 of the Millennium Development Goals (MDGs) (8). The aim is to reduce the maternal mortality rate by 75% by 2015. Since maternal mortality is costly to measure and professional attendance at delivery is assumed to reduce maternal mortality (9), the proportion of deliveries with a professional or skilled attendant is used as a progress indicator (10). Although the mid-term assessment of this objective revealed considerable progress made in some areas, improvement has been poor in sub-Saharan Africa, and significant variations persist between developed countries and developing countries where the maternal mortality ratios are nine and 905 per 100,000 livebirths respectively (11). Moreover, in rural areas, the limited use of maternal care and assisted childbirth is crucial, further increasing the maternal mortality ratio.

We should mention that full access to healthcare and the best use of ANC are not enough. Women who consult health workers during their pregnancy often do not give birth in a health centre, particularly in rural areas. Hence, it is necessary to consider the sociocultural barriers, the accessibility of ANC, and any other reasons for the limited use of obstetrical services by women. Several studies in both rural and urban areas have addressed the sociocultural barriers to the use of health services during pregnancy and childbirth (11, 12) but relatively few studies have dealt with the factors relating to health facilities themselves and to the quality of services provided. The problem of the quality of prenatal care is, thus, of great current concern for different stakeholders in the health sector in developing countries.

Burkina Faso, like other countries, has developed a number of programmes to implement initiatives for safe motherhood, namely the promotion of prenatal services, supported by guidelines establishing the standards and procedures of health services to promote maternal and child health. The rate of the use of these services is low, despite the availability of medical facilities, even if progress has been recorded in recent years (13–16). During 1998–2003, the ANC attendance rate rose from 57% to 70% in rural areas while it remained stable (96% vs 97%) in urban areas (17, 18). On the other hand, the rate of childbirth in the home remained too high and decreased only from 67% to 61% during 1998–2003 in Burkina Faso as a whole and from 74% to 68% in rural areas (17, 18). However, 36% of women who gave birth in the home had benefited from at least one ANC consultation during their pregnancy (18).

The aim of this study was to identify primary healthcare facility and antenatal care characteristics predictive of an assisted delivery in rural Burkina Faso.

## MATERIALS AND METHODS

### Setting

The study was conducted in the health district of Bogandé, Gnagna province in the Eastern region and listed among the priority provinces in the poverty-reduction strategic paper for Burkina Faso. The district, covering 8,640 sq km, has around 350,000 inhabitants. The major ethnic group is Gourmantché (74%), and the economy primarily depends on agriculture and animal breeding. The district has 25 primary health centres that provide all the available maternal and child healthcare services and a hospital of reference with an advanced surgical unit. However, the health coverage remains modest with one health centre for, on average, 11 villages and 14,000 inhabitants. ANC is provided two or three days a week and is led by a birth assistant or a nurse and according to the national procedure. The programme includes a talk about health, the assessment of pregnant women by listening to their history, a physical examination, laboratory tests, the provision of tetanus toxoid immunization, iron/folate supplementation, and prophylaxis for malaria. The health talk is intended to cover nutrition, malaria, sexually transmitted infections/HIV/AIDS, danger-signs during pregnancy and delivery, family planning, breastfeeding, and care of the newborn. Staff members at these facilities receive additional training to enable them to provide these services.

On registration of a pregnant woman, a monitoring sheet is maintained until childbirth. This sheet records the results of each examination pursuant to the standards and procedures recommended at the national level. Nevertheless, the attendance rate in antenatal care in this district remains one of the lowest in the country.

### Study design and data collection

In November 2003, we conducted a cross-sectional study of all primary healthcare facilities (n=22) in Bogandé district that had been operational for at least one year. A visit to each health centre helped list the materials, equipment, and all the posted and active staff, including their training skills on specific reproductive health topics. The physical check helped assess the state of tools and obstetrical equipment, and measurement equipment was checked against the standards for reliability. Analysis of functioning of the health centres was undertaken to assess their ability to provide reproductive health services according to the standards established in Burkina Faso, based on the following criteria:

- Availability of specific and non-specific equipment, drugs and reagents, and data-collection tools at the facility;
- Availability of a sufficient number of qualified staff; and
- Training experience of the working staff on prevention of infection, family planning, use of partogramme, obstetrical and neonatal emergency care, and breastfeeding.

A scoring system was established to calculate: (a) an equipment score (total number of working equipment and tools available in good condition), (b) a staff score (total number of the following categories: senior midwife and senior obstetric assistant, senior nurse, junior nurse, birth assistant, and junior health workers, and (c) a training score (total number of people trained in the following topics: infection prevention (IP), family planning (FP), use of the partogramme, obstetric and neonatal emergency care (ONEC), and breastfeeding counselling.

A non-participating observation of five consecutive antenatal consultations, undertaken by the same healthcare provider on the day of our visit was made in each health centre, by the same observer (a medical doctor). The quality of services was assessed based on the national standards for all the operations, attitudes, and questions put to the pregnant woman during ANC. This standard includes five dimensions: (a) Components of reception (n=7, such as including hearing discretion, visual discretion, and incentive measures for the patient); (b) Types of information collected (n=7, such as marital status, antecedents, way of life, vaccination status, record of current pregnancy, etc.); (c) Components of clinical examination (n=16, such as all stages of a complete clinical examination); (d) Components of gynaecological examination (n=20, such as all stages of a complete gynaecological examination and care relating to the prevention of infections); and (e) Components of decision-making (n=21, such as advice given to the woman, therapeutic decisions, regulations, etc.). Each component of these five dimensions of the standard was scored from 0 to 2: 0 if not carried out, 1 if passably carried out, and 2 if correctly carried out. The scores were then added up and averaged out of 20 to determine a score for each stage of ANC. The highest possible scores were 14 for reception and dialogue, 32 for clinical examination, 40 for gynaecological examination, and 42 for decision-making. Finally, during a scheduled interview with the team leader of each maternity facility, a semi-structured questionnaire was used for assessing the difficulties faced in the implementation of reproductive health activities. We also noted on the register of each health centre the number of childbirths during one full year of the operation, the number of antenatal consultations for each childbirth, and demographic information on the health district.

### Statistical analysis

Data collected were entered using the EpiData softwere (3.0 version) (19) and analyzed using the Info EPI software (version 6.04d) (20) and the SAS System (21). A principal component analysis was performed on all the afore-mentioned scores (scores relating to equipment, staff, training, and various scores of ANC) to examine the relationship between these primary scores to construct more synthetic scores, grouping the primary scores in the same dimension. Not surprisingly, analysis grouped the scores in two categories: equipment, staff, training in one category and scores connected with ANC in the other. Finally, adding up the scores in each category gave two total scores: (a) a total score for the provision of care, i.e. the sum of the scores for equipment, staff, and training, and (b) a total score for the quality of ANC (provided it conformed to the recommendations of the Ministry of Health), i.e. the sum of the scores for different stages of ANC.

For each health centre, the obstetrical coverage rate was calculated by the ratio of the number of assisted childbirths over the year and the number of expected childbirths for the same year, based on the size of the population covered in the health district. Kendall's non-parametric test was used for studing the relationships between the obstetrical coverage rates: the score for the provision of care on the one hand and the quality of ANC consultation on the other.

## RESULTS

**Fig. 1.**
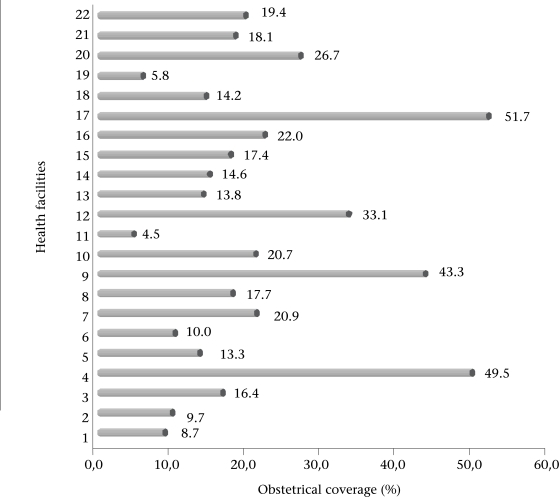
Distribution of obstetrical coverage by health facility

The professional childbirth rates (obstetrical coverage) ranged from 4% to 51% depending on the location of the health centre, with an average of 20±14% for the whole district. Figure 1 shows the obstetrical coverage rate of the 22 health centres. Nearly 95% of the assisted childbirths (n=3,479) were pregnancies that had benefitted from at least one ANC consultation. The average number of antenatal consultations per assisted childbirth was 2.6.

**Fig. 2.**
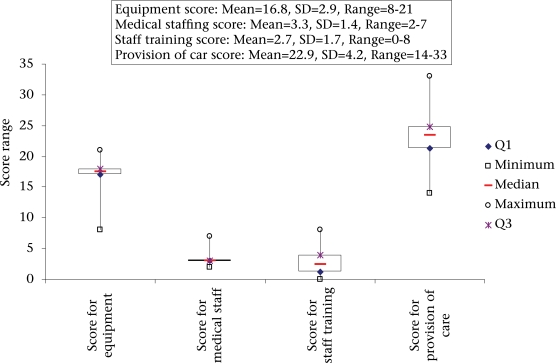
Breakdown of the total score for provision of care into its components: equipment, medical staff, and staff training

The physical checking of the material and equipment available in each health centre revealed problems at all the health centres but with a major variation between centres (scores ranging from 8 to 21 out of a maximum of 24, with an average of 16.8). No health centre had equipment to measure the length of the child. Figure 2 shows the details of the equipment score.

SD=Standard deviation

The average number of health workers per health centre was three; this was the number of staff members in 18 of the 22 maternity centres. Only three health centres had the theoretical minimum number of four health workers recommended nationally. Figure 2 shows the details of the score for medical staff. In addition, 45% of the health centres had no junior birth assistants. Concerning the training of staff in the specific topics of reproductive health, for the whole district, an average of 1.6 agents per centre had been trained in the prevention of infection, 1.5 in family planning, 0.3 in the use of Partogramme, 0.1 in breastfeeding, and none in obstetrical and emergency care for neonates. The final score in staff training was, thus, very low, with only two health centres having at least a person trained in four of five topics. Figure 2 shows the details of the staff training score.

The total score for the provision of care ranged from 14 to 33 depending on the health centre (with a maximum of 38). Only 30% of the health centres had an above-average total score for the provision of care. Figure 2 shows the details of this score.

### Assessment of ANC procedure

Of the 22 health centres visited, in five cases, no woman was receiving an antenatal consultation on the day of our visit and, for logistical and temporal reasons, we were unable to return for a second visit. Thus, only 17 health centres were checked for ANC; in some of them, there were fewer than five women attending ANC on the day of our visit; in all, 81 antenatal consultations were observed.

**Fig. 3.**
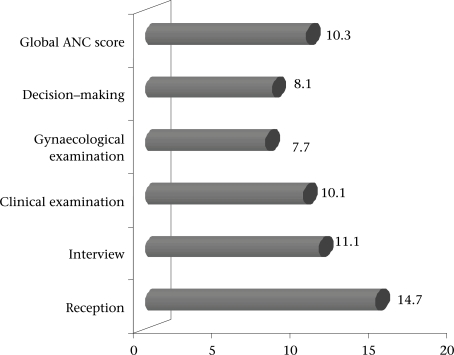
Mean scores for global ANC and each stage of ANC (each score ranges from 0 to 20)

A birth assistant or a matron carried out the antenatal examination in 44%, a senior nurse in 25%, a junior health worker in 19%, and a nurse in 12% of the cases. The observation of ANC revealed failures at all the stages but especially at the level of gynaecological examination, decision-making, and clinical examination. Figure 3 shows the average scores out of 20 for each stage of ANC. For 73% of the 81 observations, the total score was below average. More precisely, overall reception was acceptable. However, some questions were asked in the waiting-room and were generally incomplete; the information that was lacking most often was the way of life (61%), personal background (59%), vaccination status (43%), and the record of current pregnancy (37%). Clinical examination began by recording weight, height, and blood pressure which was generally done in groups in the waiting-room. Physical examination was performed individually in all the cases and was conducted in a room used as an office equipped with an examination table. The light was poor in all the cases; daylight was the only source in 78% of the cases. Clinical examination was often interrupted by a visitor or another health worker or even a stranger, which did not guarantee the confidentiality of information. The examination was brief in all the cases and cardiopulmonary sounding was not performed in any case. A speculum was not used for gynaecological examination in any health centres. Vaginal examination was often carried out using a different fingerstall for each patient but using the same pair of gloves for all the patients. Hands were washed with soap before and after each examination in a very few cases where water was available.

ANC=Antenatal care

After the physical examination, a very few women were informed of the results and the course of their pregnancy. For the women close to term, the location of childbirth was seldom discussed, and signs of alert were also not properly discussed. Most risk factors were not considered in an active way, and women at risk did not benefit from any particular care. The search for albumin and sugar in urine was carried out in only 9% of the cases. Other obligatory complementary examinations were required only in the reference maternity centre of the district; serology toxoplasmosis and echography were not requested at all. Anti-tetanus vaccination was suggested in all the cases when the woman was not properly vaccinated or had no vaccination record. Concerning the prevention of disease, all the women were benefited from a prescription for chloroquine for malaria and iron+folic acid for anaemia. The dose was specified in all the cases but less was said about the importance of these regulations to the women. On the whole, women were not correctly involved in the process; they received very little individual advice on pregnancy hygiene, food hygiene (30%), and planning of childbirth during ANC meetings. However, at the majority (n=18) of the health centres, days when antenatal consultations were held started with a group-discussion session on a reproductive health topic with visual aids.

### Interviews with health workers

The difficulties faced in the provision of good-quality ANC mentioned by the health workers were practically the same for all the health centres. These were poor attendance at the health centres, the delay in the use of prenatal care by women (generally in the second or third trimester), or even only in the case of an emergency health problem, the weak observance of anti-malaria chimioprophylaxis and anti-anaemics, and the refusal of some women to be examined by a male health worker.

### Relationship among obstetrical coverage, score for provision of care, and quality of antenatal consultation

There were no correlations between the classification of the structures by rank of care provision and obstetrical coverage (tau of Kendall=0.03, p=0.81). Some health centres with a weak obstetrical coverage rate still ranked well for the provision of care; in the same way, some health centres had a relatively-good obstetrical coverage rate, despite their low score for the provision of care. The results of analysis of the classification according to the quality of ANC and the rank of obstetrical coverage showed that the obstetrical coverage rate tended to follow the quality of ANC (tau of Kendall=0.24, p=0.18).

## DISCUSSION

The obstetrical coverage observed in the district of Bogandé was 20% whereas it was 31% for all rural areas in Burkina Faso (18). There was also a significant variability among the health centres. However, the two maternity centres with the highest rates of obstetrical coverage (49% and 51%) are supported by religious orders and, thus, benefit from more appropriate means, and attract patients from areas far beyond their geographical health district. Except in these two cases, the maximum coverage rate was 43%, and the minimum was 4%, which slightly reduced the variability between the health centres. This rate of professional childbirth is low, below the average in developing countries. Four in five study women had given birth in the home. This high rate of childbirth in the home gives a glimpse of the high rate of maternal mortality in the district and means that the Objective 5 of MDGs is unlikely to be reached. Unfortunately, we were not able to estimate the maternal mortality rate in this study because of its retrospective design, the weakness of the health information system, and the resulting absence of systematic identification and recording of maternal deaths.

This weak obstetrical coverage is probably, to some extent, related to the same factors found in other studies, such as geographical and financial accessibility and health facilities (22–25), socioeconomic barriers, or the educational level of women (26). Indeed, in most developing countries, charges for health services have to be paid for directly. According to a recent study in three African countries, 92% of women in Burkina Faso paid some fee for their previous professional delivery, and the cost was relatively high (27). For example, a complicated delivery represents more than 16% of the mean monthly household income in Burkina Faso (27). This is all the more probable as the socioeconomic and educational levels of the women were very low in the medical district of Bogandé where only 7% of the girls attended school and the economy is based on animal breeding and agriculture; the area still faces cereal shortage due to the poor quality of the soils and the climate (28).

However, these factors alone could not explain why so many women, who had attended antenatal consultations, did not use assisted childbirth. Our initial assumptions were on the role of other factors, particularly the provision of care and the quality of ANC. It was shown that the lack of qualified staff, poor management of existing staff, bad allocation of limited resources, bad relationships between health workers and pregnant women, and the lack of tools account for the unsatisfactory provision of maternal care, which is decisive for a good obstetrical coverage (29). The set of the scores for health staff, training of staff, equipment, and skills in reproductive health was weak in our study. We found no significant correlation between the different scores for the functioning of health services and the obstetric coverage. We could question our methodology for the measuring equipment and the provision of care and particularly the fact that the total score combines different aspects (equipment, staff, and training). However, when tested independently, the three corresponding scores did not show any further links with the obstetrical coverage (results not shown). We can, thus, only make assumptions to explain this result, including the fact that the provision of care is simply not a key factor in this underprivileged context where tradition plays a significant role. We could also assume that the overall provision of care is weak in the province and that the situation is too homogeneous for the role of care provision to be revealed, despite the relative differences between the health centres.

The main reason for the low obstetrical coverage could be the quality of care provided in the antenatal period. This is recognized as a determining factor in the use of health services (25). One study on the factors that influence the health facilities chosen at delivery has shown that the women's choice of facility is based on the quality of previous experience and on her trust in health workers at the facility (30). Like the results of previous studies in other countries (4) and also of other studies in Burkina Faso (14, 16, 31), we recorded failures at all the stages of ANC. We did not find a significant link between the obstetrical coverage and the quality of care but there was a trend. Our appreciation of the quality of care may have been too limited because we were only able to observe only one session per maternity centre, i.e. only one person was observed at each centre, and because, in some maternity centres, no women or only a few women were present. We can, thus, suppose that the non-attendance of ANC by pregnant women is related to the poor quality of services, thus introducing a selection bias. Despite this possible bias, some elements of our observations could help explain the weak use and even the abandonment of health services by the women. The problem of the quality of reception is probably not to blame as it was the most satisfactory point in all the stages of ANC observed, which is often the case in rural areas (32), in contrast to what is generally observed in urban areas (31, 33). On the other hand, the failures of other stages of ANC, particularly decision-making and gynaecological examination, are probably involved. The lack of screening and the lack of information on the risks of pregnancy, childbirth, and possible treatment that can be provided to patients, and the poor exchanges on planning of childbirth may account for the lack of use of medical facilities for childbirth (22). Moreover, in Burkina Faso, during the demographic and health survey in 2003, women declared that the individual attention that they receive encourages them to continue with their antenatal follow-up, although they also suffered from the lack of information on the signs of risk during pregnancy and the absence of planning of childbirth (18).

The main aim of this work was to assess the factors relating to the functioning of health centres to be able to explain the low use of health services at delivery by the women in contrast to the high use of antenatal care services. Our main conclusion is that the ability of the primary health centres in a rural district in Burkina Faso to provide good antenatal care is low. The key factors involved in the limited use of professional childbirth relating to maternal health services are the quality of antenatal care. Investing in the improved quality of maternal care in the primary health facilities may increase the number of professional deliveries and improve the effectiveness of health facilities in providing facilities for professional delivery.

## ACKNOWLEDGEMENTS

The authors thank the UR106 of the Institute of Research for Development for the funding and its assistance with refining the finished document and also the Bogandé district team for its permission to carry out this research.
